# Migrate3D: Software for simplified post-tracking analysis of 3D and 2D cell migration data

**DOI:** 10.21203/rs.3.rs-2451513/v2

**Published:** 2024-01-18

**Authors:** Emily R. Mynar, Matthew W. Kinahan, Markus Thali, Menelaos Symeonides

**Affiliations:** University of Vermont; University of Vermont; University of Vermont; University of Vermont

**Keywords:** cell migration, cell biology, cell motility, quantitative biology, bioinformatics, python, principal component analysis, machine learning

## Abstract

Migrate3D is a cell migration analysis tool whose purpose is to computationally process positional cell tracking data generated via other image acquisition/analysis software and generate biologically meaningful results. The functionalities of Migrate3D include step-based calculations of each cell track, single-cell-level summary statistics, mean squared displacement analysis, and machine learning-based evaluation of the entire dataset and subpopulations of cells found within it. The parameters calculated within Migrate3D have been previously developed and validated by other groups, and were selected to facilitate extraction of the maximum depth of information possible from input datasets. Variables are user-adjustable to enable customized analyses of diverse motility patterns and cell types, both in three-and two-dimensional timelapse data. Independent of any particular upstream image analysis or cell tracking software, Migrate3D only needs positional data over time to execute the suite of calculations. This presents a unique opportunity to standardize and streamline cell migration analysis.

## 1. Introduction:

Migration is a critical cell behavior in a wide array of physiological, developmental, and disease-related processes (SenGupta *et al*. 2021). Quantitative analysis of cell motility can help detect unique migration patterns in cells of interest (e.g. virus-infected cells, cells bearing a gene mutation, or cells of a particular lineage) and elucidate mechanisms underlying related phenotypes (Georgantzoglou *et al*. 2022; Jerison and Quake 2020; Mrass *et al*. 2017; Roy *et al*. 2020). More recently, such analysis has been employed together with machine learning to help identify cell subsets within complex populations of migrating cells (Chan *et al*. 2020; Kesapragada *et al*. 2022).

Modern time-lapse microscopy techniques in tandem with computational image processing have allowed for high-throughput tracking of cells *in vivo* and *in vitro* (Masuzzo *et al*. 2016). A typical workflow for cell migration experiments involves sample preparation, image acquisition, cell segmentation, segment-based cell tracking, and finally track analysis (Masuzzo *et al*. 2016). Many software tools are available for image processing, including Imaris (Oxford Instruments), CellProfiler (Stirling *et al*. 2021), TrackMate (Ershov *et al*. 2022), and many others (Masuzzo *et al*. 2016). However, open-source and customizable analysis tools downstream of image processing and acquisition are needed (Schienstock and Mueller 2022). Those that are available are often experiment-specific, restricted to data generated from specific software, or require signicant programming knowledge to operate. The lack of a universal program that is user-friendly and able to analyze migration data from many sources has been identified as a major hurdle to cell migration research (Svensson *et al*. 2018).

Here, we introduce Migrate3D, a Python program that streamlines and automates cell migration analysis in three- and two-dimensions and calculates useful motility parameters previously identified as biologically relevant (reviewed in (Beltman *et al*. 2009)). Migrate3D analyzes individual cells in a dataset between timepoints (step-based) and over the cell’s entire tracking period (cell-based) and is complete with a graphical user interface (GUI). Finally, embedded in Migrate3D is the ability to perform dimensionality reduction by unsupervised machine learning, namely using principal component analysis (PCA), using the calculated parameters. Migrate3D presents an opportunity to standardize cell migration analysis independent of image processing software and calculate parameters that are the most useful when analyzing cell movement.

## 2. Program Description

### 2.1. Program Description:

Migrate3D was developed in Python and compatible with Python versions >= 3.9, and the GUI is built with the dearpygui library. To use Migrate3D, the input needs to be in comma-separated value (*.csv) format, and each row must include a unique cell identifier (ID), X/Y/Z coordinates, and the time. In other words, each row must represent the position of one cell at one point in time. Migrate3D has five major functionalities: data formatting, step-based calculations, cell-based summary statistics, contact detection, and principal component analysis (PCA). Several variables are adjustable in order to accommodate different types of motility or different research questions. Upon completion of a run, Migrate3D returns an Excel workbook (*.xlsx) with multiple worksheets displaying the results.

### 2.2. Data Formatting:

All data formatting functions leave the original input dataset intact, and instead export the reformatted dataset alongside the results in an Excel workbook. While uninterrupted tracks are ideal, Migrate3D can interpolate missing data if needed, as long as the different segments of the track belong to the same unique cell ID. Similarly, if cells are multiply tracked such that there are multiple X/Y/Z coordinates for a single timepoint (usually resulting from segmentation errors), as long as these segments are assigned to the same cell ID (usually achieved by manual curation of the data), Migrate3D will average them into one position for that timepoint. Migrate3D is also able to handle two-dimensional data, and it provides the option to convert three-dimensional data into two-dimensional data by ignoring the Z position.

### 2.3. Calculations and Summary Statistics:

Step-based calculations are performed to extract the most information possible from a track. Instantaneous displacement, total displacement, path length, instantaneous velocity, instantaneous acceleration, point-to-point Euclidean distance, and relative turning angle are calculated (Beltman *et al*. 2009). A number of user-input variables are utilized in these calculations; these include the time lag (τ or tau) and the minimum displacement limit. Euclidean distance and relative turning angle are calculated over a given τ value such that these parameters can be tuned to be measured at smaller or larger time intervals according to the particular type of sample. Euclidean distance and relative turning angle are filtered based on the per-timepoint minimum displacement limit variable so that any background nonspecic movement can be omitted. Cell-based summary statistics will also be calculated for all step-based parameters including displacement ratio, outreach ratio, arrest coefficient, and mean squared displacement (MSD). Average displacements and standard deviations are also reported per tau value across the entire dataset, as well as for cell subsets within the dataset (if provided by the user in a ‘categories’ file; see PCA section).

### 2.4. Contact Detection:

If enabled, the Contacts process will iterate over all the cells in the dataset and compare their X/Y/Z position at each timepoint. If the intercellular distance at a given timepoint is lower than the contact length limit set by the user, it will be recorded as a contact for the two cells in question. The resulting dataset is also further filtered down to exclude cell divisions (where the daughter cells are, for some time right after cytokinesis, in close proximity but not because a new cell-cell contact has formed) and any contacts involving non-motile or ‘dead’ cells (whether or not they are truly dead). Cell divisions are detected by evaluating the unique identifier of each pair of cells that are found to be in contact, and if those identifiers differ from each other by exactly one, they are considered to be daughter cells. This may limit the universality of the function and may require some manual reformatting of data to properly utilize it. A cell is determined to be non-motile if its arrest coefficient is higher than a user-set minimum (note that the arrest coefficient is, in turn, based on the minimum displacement limit set by the user). Finally, a summary of each individual cell’s contact history within the dataset is also produced, including the number of contacts made, the total time spent in contact, and the median contact duration.

### 2.5. Dimensionality Reduction (PCA):

If a dataset contains known cell subsets (e.g. virus-infected and uninfected cells thanks to a fluorescent reporter), the user has the option to provide a ‘categories’ file (also a CSV), where each cell ID has been annotated with a category (which can be any string or value). If this is done, Migrate3D will use the migration parameters described above to perform dimensionality reduction by PCA and subsequent statistical analyses comparing the given cell subsets. A Kruskal-Wallis test and a Dunn post-hoc analysis are performed on the PCA results based on the provided categories in order to evaluate whether significant differences exist between known subsets of cells. A separate Excel workbook output is generated for the results of PCA.

## 3. Conclusions and Limitations:

Migrate3D is a migration analysis tool that is able to analyze cell motility data generated using different image processing and tracking software packages. We developed Migrate3D to fulfil our own need for a user-friendly, GUI-based tool which accommodates diverse datasets thanks to user-adjustable variables. We will present results from our own analyses done using Migrate3D in future publications. Further, we have analyzed publicly available datasets (LaChance *et al*. 2022b) and compared our results to the associated published analyses (LaChance *et al*. 2022a) to confirm that the calculations done within the code are correct (data not shown).

Limitations of Migrate3D include: an inability to detect mitotic events within a cell track without manually giving the daughter cells’ tracks unique cell IDs (which could lead to incorrect conclusions if not properly handled before running the program); reliance on the proper upstream management of the dataset, including rigorous sample preparation and data acquisition, proper segmentation and tracking, and potentially some manual curation of the data (as described above). However, such manual curation is, in our view, fundamental to understanding one’s own data and producing scienti cally-sound findings.

Migrate3D is an open-source software package available freely at https://github.com/msymeonides/Migrate3D/. Detailed instructions for installation and operation are included therein. Our hope is that Migrate3D will be widely adopted and further developed by others to help establish a universal standard in cell migration analysis.

## Availability and Implementation:

Migrate3D is an open-source software suite implemented in Python (version 3.x) and functions on Windows, MacOS, and Linux systems. Source code and detailed installation and usage instructions are available freely at:
